# Patient access to reimbursed biological disease-modifying antirheumatic drugs in the European region

**DOI:** 10.1080/20016689.2017.1345580

**Published:** 2017-07-05

**Authors:** Zoltán Kaló, Zoltán Vokó, Andrew Östör, Emma Clifton-Brown, Radu Vasilescu, Alysia Battersby, Edward Gibson

**Affiliations:** ^a^ Department of Health Policy and Health Economics, Eötvös Loránd University, Budapest, Hungary; ^b^ Rheumatology Clinical Research Unit, Addenbrookes Hospital, Cambridge, UK; ^c^ Global Health & Value, Pfizer Ltd, Tadworth, UK; ^d^ Wickenstones, Oxfordshire, UK

**Keywords:** Biological disease-modifying antirheumatic drugs (bDMARDs), rheumatoid arthritis, reimbursement, biosimilars

## Abstract

**Background & Objectives**: Biological disease-modifying antirheumatic drugs (bDMARDs) for the treatment of rheumatoid arthritis (RA) are not always accessible to all patients in accordance with international guidelines, partly owing to their high direct costs against a background of restricted healthcare budgets. This study compares the size of RA patient populations with access to reimbursed bDMARDs across 37 European countries, Russia, and Turkey, according to their treatment eligibility defined by European League Against Rheumatism (EULAR) recommendations and national reimbursement criteria.

**Methods**: The size of the RA patient population eligible for bDMARD treatment was estimated in a population model using published RA epidemiological data and clinical criteria defined by 2013 EULAR recommendations along with national reimbursement criteria defined in a survey of the 39 countries in November 2015.

**Results**: According to EULAR recommendations, 32% of the total RA population in the European region is eligible for bDMARD treatment. However, only an average 59% of this EULAR-eligible population remains eligible after applying national reimbursement criteria (from 86% in ‘high access’ to 13% in ‘low-access’ countries).

**Conclusion**: Access to reimbursed bDMARDs remains unequal in the European region. As biosimilars of bDMARDs are introduced, changes in reimbursement criteria may increase access to bDMARDs and reduce this inequality.

## Introduction

Rheumatoid arthritis (RA) is a chronic, systemic autoimmune disease that manifests as joint pain and stiffness and the progressive destruction of joints. The worldwide prevalence of RA is estimated at 0.24%, with at least twice as many women affected as men [1]. RA causes considerable functional disability and accounts for 0.8% of all disability-adjusted life-years lost in Europe [2,3]. Effective drugs are increasingly available to reduce disease activity and prevent joint deformity in RA. To help clinicians to make treatment decisions faced with the abundant therapeutic options available, yet often insufficient information on differential efficacy and safety, the American College of Rheumatology (ACR) and the European League Against Rheumatism (EULAR) have made recommendations for the management of RA with these drugs [4,5,6].

The EULAR guidelines recommend as the first treatment strategy in patients with active RA [defined as Disease Activity Score based on 28 joint count (DAS28) > 3.2] [7], conventional synthetic disease-modifying antirheumatic drugs (csDMARDs), such as methotrexate (MTX), sulfasalazine, and leflunomide with or without glucocorticoids. If a patient is intolerant of or non-responsive to csDMARDs, that is, the treatment target is not reached within 6 months or improvement is not seen at 3 months, treatment with a first biological disease-modifying antirheumatic drug (bDMARD) (in the presence of prognostically unfavourable factors such as early joint damage) or another csDMARD (in the absence of prognostically unfavourable factors) is recommended. If the first biological treatment strategy fails, any other bDMARD may be used. Targeted synthetic disease-modifying antirheumatic drugs (tsDMARDs) are currently recommended if the treatment target is not achieved with the first csDMARD strategy, and when poor prognostic factors are present; in such patients, addition of a bDMARD or a tsDMARD should be considered and current practice would be to start a bDMARD. The bDMARDs include the tumour necrosis factor inhibitors etanercept, adalimumab, certolizumab pegol, infliximab, and golimumab; the T-cell costimulation inhibitor abatacept; the anti-B-cell agent rituximab; the interleukin-6 receptor-blocking monoclonal antibody tocilizumab; and the interleukin-1 inhibitor anakinra. The tsDMARDs include tofacitinib and baricitinib, synthetic DMARDs specifically designed to target janus kinases.

The availability of bDMARDs for the treatment of RA has improved the ability to control disease activity [8]. However, bDMARDs are not always reimbursed for all patients who are recommended for treatment by EULAR guidelines (with a DAS28 > 3.2 and after failure of two or more csDMARDs), partly owing to their high direct costs against a background of restricted healthcare budgets. The high cost of bDMARDs has meant that macroeconomic conditions may negatively influence patient access to reimbursed treatment in some regions of Europe [9], with lower income countries having poorer access to RA treatments [10]. A 2014 study of national criteria for bDMARD reimbursement in RA in 46 European countries showed that most countries did not reimburse bDMARDs in line with EULAR guidelines. Instead, more stringent national reimbursement criteria are imposed [11].

Against the background of unequal access to reimbursed bDMARDs in the RA patient population in the European region (defined in this study as 37 European countries, plus Russia and Turkey), the objective of this study was to compare the theoretical sizes of the RA patient populations with and without access to reimbursed bDMARDs on the basis of EULAR criteria and national reimbursement criteria, and to raise awareness of the current gaps in patient access to bDMARD treatment in the European region.

## Methods

### Calculation of bDMARD-eligible patient population based on EULAR criteria

A population model was developed to estimate the size of the RA patient population eligible for bDMARDs using treatment categories defined by EULAR guidelines (all RA patients with a DAS28 > 3.2 and two or more csDMARD treatment failures) and national reimbursement criteria previously described by Putrik et al. [11]. The categories are: the minimum number of failed csDMARDs, the minimum disease duration before the start of therapy, the minimum disease severity (disease activity), the time-point chosen to assess the response, and the stopping rules (Table 1). The model calculates the prevalence of a patient population per category using prevalence figures reported in representative patient registries and clinical and observational studies (Table 1).

**Table 1.** Disease categories and their subcategories used in recommendations and criteria for biological disease-modifying antirheumatic drug (DMARD) eligibility and the assumptions used to predict their prevalence.

Category	Sub-category	Percentage of total RA population defined by the restriction	Source of prevalence data
Failed csDMARDs	< 2	56.9%	Aletaha 2002 [12]
	2–4	34.2%	
	≥ 5	2.7%	
Minimal disease duration	≤ 6 months	1.9%	Humphreys 2013 [13]
	> 6 months	98.1%	
Disease activity	DAS28 ≤ 3.2	25%	Sokka 2007 [14]
	DAS28 > 3.2 and ≤ 5.1	60%	
	DAS28 > 5.1	15%	
Time-point to assess response	< 12 weeks	81%	Kavanaugh 2008 [15], Hetland 2010 [16]
	12–24 weeks	88.1%	
	> 24 weeks	86.8%	
Stopping rules	DAS28 > 1.2	42%	Hetland 2010 [16]
	DAS28 > 0.6 and ≤ 1.2	39%	
	DAS28 ≤ 0.6	19%	

csDMARDs, conventional synthetic disease-modifying antirheumatic drugs; DAS28, Disease Activity Score based on 28 joint count; RA, rheumatoid arthritis.

The prevalence figures used by the model are: 56.9%, 34.2%, and 2.7% for the minimum number of failed csDMARDs fewer than two, two to four, and five or more, respectively [12]; 1.9% and 98.1% for a minimum disease duration at the start of therapy of ≤ 6 months and > 6 months, respectively [13]; 25%, 60%, and 15% for minimum disease activity DAS28 ≤ 3.2, DAS28 > 3.2 ≤ 5.1, and DAS28 > 5.1, respectively [14]; 81%, 88.1%, and 86.8% for the time-point to assess the response < 12 weeks, 12–24 weeks, and > 24 weeks, respectively [15,16]; and 42%, 39%, and 19% for stopping rules DAS28 > 1.2, DAS28 > 0.6 ≤ 1.2, and DAS28 ≤ 0.6, respectively [16] (Table 1).

**Figure 1.**
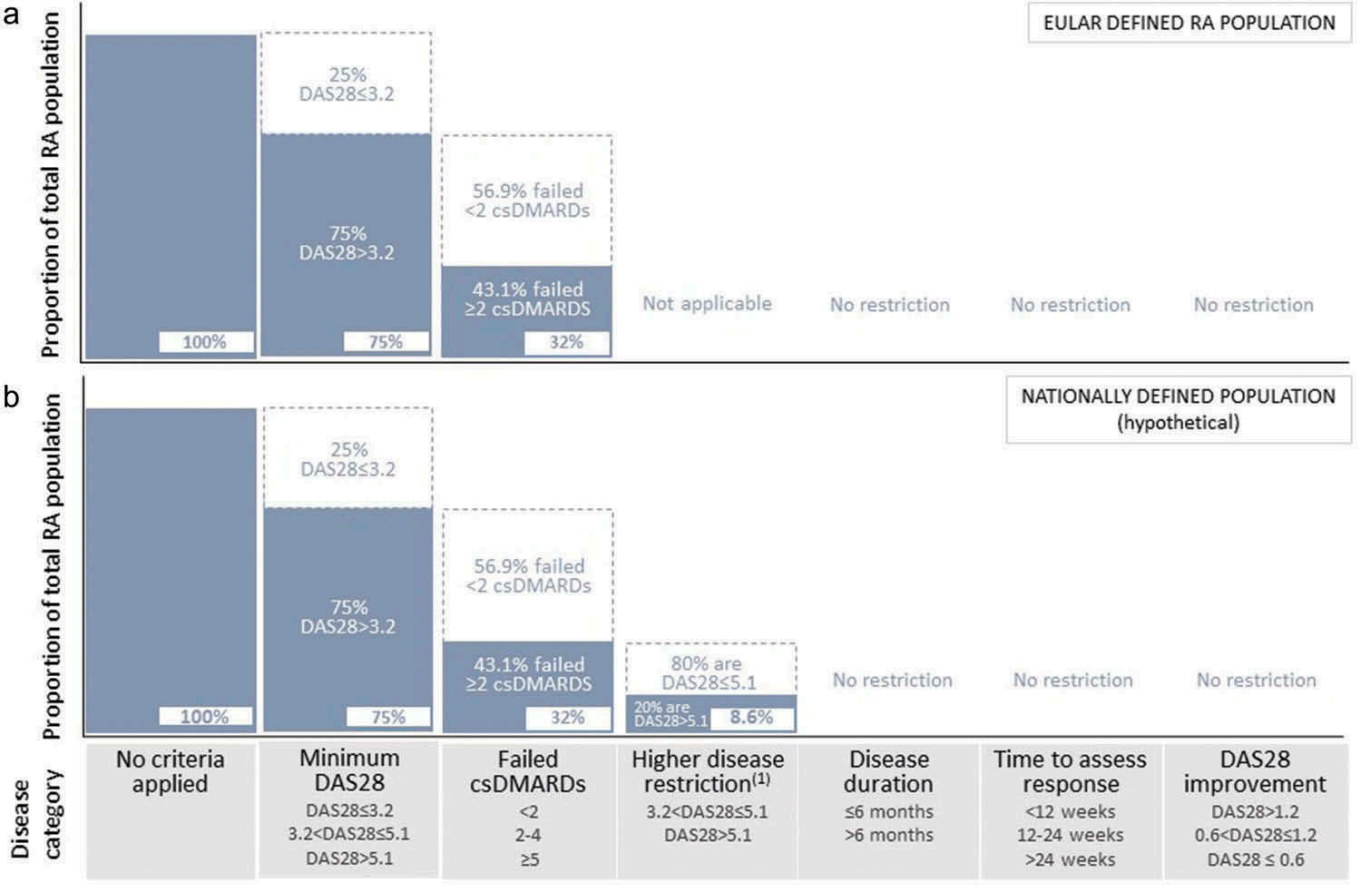
Method used by the population model to calculate populations defined by disease categories. (A) Schematic showing how the model calculates the proportion of the total rheumatoid arthritis (RA) population defined by European League Against Rheumatism (EULAR) recommendations [Disease Activity Score based on 28 joint count (DAS28) > 3.2 and two or more failed conventional synthetic disease-modifying antirheumatic drugs (csDMARDs)]. The model first removes patients with a DAS28 ≤ 3.2 (25% of the total RA population, see Table 1 for model assumptions) from the total RA population. Next, the model subtracts the RA population with fewer than two failed csDMARDs (56.9%). This leaves 32% of the total RA population that is eligible for bDMARD treatment according to EULAR guidelines. (B) Schematic showing how the model calculates the proportion of a hypothetical country’s RA population defined by national reimbursement guidelines that specify two or more failed csDMARDs and a DAS28 > 5.1. Continuing from the EULAR-defined 32% of the total RA population (as per calculations performed in part A of this figure), the model removes DAS28 ≤ 5.1 patients (80% of the RA population, see Table 1 for model assumptions) to generate 8.5% of the total population. ^(1)^ The higher disease restriction applies to national disease severity criteria that are more stringent than the EULAR recommendations of DAS28 > 3.2.

The size of a selected country’s eligible RA population is calculated by multiplying the country’s total population [17] by its prevalent RA population (see Table S1 in the supplementary material) and by the proportion of patients who fall into the defined categories (Figure 1). For multiple criteria (such as the criteria DAS28 > 3.2 and two or more csDMARD failures in the EULAR guidelines), the model defines the proportion of patients in one criteria as a proportion of patients in another, as in the sequence depicted in Figure 1. Using the two criteria of the EULAR guidelines as an example, the proportion of patients defined by EULAR guidelines (DAS28 > 3.2 and two or more csDMARD failures) is 32%, and is calculated by multiplying the proportion of patients in the two or more csDMARD failures category (43.1%) by the proportion of patients in the DAS28 > 3.2 category (75%) (Table 1).

### Calculation of bDMARD-eligible patient population based on national reimbursement criteria

A questionnaire was developed based on the clinical criteria for recommended use of bDMARDs, as defined by EULAR and outlined in Table 1. The questionnaire was completed by one or more local representatives of Pfizer Inc. from each of the 37 European countries, Russia, and Turkey in November 2015. Answers were based on representatives’ working knowledge of current national reimbursement criteria. In addition to clinical access criteria, the questionnaire explored national criteria regulating the diagnosis and initiation of treatment (who can prescribe bDMARDs, what the requirements are to start the first biological drug), criteria for assessing bDMARD response (time to assessment), rules for stopping bDMARDs, and rules for switching to a different bDMARD. The total number of patients eligible for treatment according to national reimbursement criteria was then calculated in the same way as described above for the proportion eligible under EULAR guidelines and outlined in Table 1 and Figure 1.

### Composite eligibility score

In addition to calculating the number of patient eligible for bDMARDs under national reimbursement criteria, each country was assigned a composite eligibility score comprising the sum of scores (ranging from 1 to 5; a higher score meaning less restrictive bDMARD access) involving the disease duration [any requirement (0 points), no requirement (1 point)], number of csDMARDs failed [more than two (0 points), two (1 point), and fewer than two (2 points)], and level of disease activity [DAS28 cut-off > 3.2 or its equivalent (0 points), DAS28 cut-off ≤ 3.2 or its equivalent (1 point), and no requirement (2 points)] criteria, as described in Putrik et al. [11].

### Correlation analysis

To investigate whether the proportion of EULAR-eligible RA patients who have access to bDMARDs according to national criteria is correlated with a country’s healthcare expenditure as a proportion of gross domestic product (GDP) or GDP per capita, we conducted a linear regression. This compared the proportion of the nationally eligible population (according to disease severity and minimum csDMARD treatment failures) as a percentage of the EULAR-defined population with the country’s percentage GDP spent on healthcare in 2013 [18] or GDP per capita in 2015 [19].

## Results

### Predicted bDMARD-eligible patient numbers defined by EULAR recommendations

The proportion of RA patients in the European region who are eligible for bDMARD treatment according to EULAR recommendations (DAS28 > 3.2 and two or more csDMARD treatment failures) is 32%, based on the assumption that 75% of the total European RA population has a DAS28 > 3.2 [14] and that 43.1% of patients will have failed two or more csDMARDs [12]. Translating this proportion into patient numbers, theoretically about 1.7 million RA patients out of the total 5.3 million RA currently living in the European region are eligible for bDMARD treatment (Figure 1).

### Patient access to bDMARD defined by national reimbursement criteria

An internal survey of experts in 39 European countries (see Table S2 in the supplementary material) found that national criteria for bDMARD reimbursement in RA differ significantly between countries and are often divergent from the eligibility criteria defined by EULAR guidelines.

**Table 2.** Proportion of countries surveyed with requirements for minimum clinical criteria for biological disease-modifying antirheumatic drug reimbursement.

Category	Sub-category	No. of countries with national criteria
Minimum DAS28	> 3.2	26 of 39
	> 5.1	16 of 39
Disease duration	> 6 months	10 of 39
Previous treatments	> 1 failed csDMARD	32 of 39
	> 2 failed csDMARDs	23 of 39
	> 3 failed csDMARDs	4 of 39
Time-point to assess treatment response	< 24 weeks	27 of 39
Minimum DAS28 improvement at 6 months	DAS28 ≥ 1.2	7 of 39

csDMARDs, conventional synthetic disease-modifying antirheumatic drugs; DAS28, Disease Activity Score based on 28 joint count.

**Figure 2.**
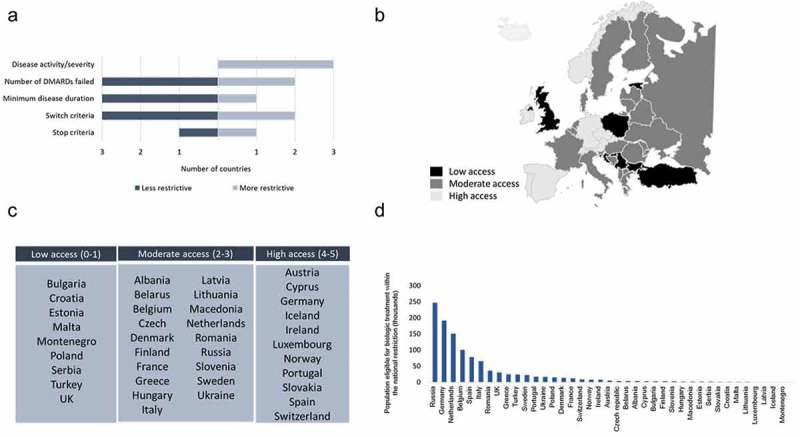
(A) Changes in national eligibility criteria reported for biological disease-modifying antirheumatic drug (DMARD) reimbursement since 2011. (B) Heat map of countries according to their access scores. (C) Grouping of countries according to low, moderate, and high access composite eligibility scores (see Methods section for definitions of low, moderate, and high). (D) Population sizes of rheumatoid arthritis patients eligible for biological DMARD treatment within national criteria.

More than two-thirds of countries (26 out of 39) require a minimum DAS28 > 3.2, and about one-quarter (10 out of 39) require a minimum disease duration of more than 6 months. Most countries require patients to have failed one or more csDMARDs, about one-third (23 out of 39) require a failure of two or more csDMARDs, and four of the 39 countries surveyed require more than three failed csDMARDs before eligibility for bDMARDs. Many countries require that treatment response is assessed at less than 24 weeks and some require a minimum DAS28 improvement of ≥ 1.2 after 6 months (Table 2). Several countries have changed their eligibility criteria since May 2011, when a previous survey by Putrik and colleagues [11] was completed, although no clear trend is apparent. The changes are summarized in Figure 2(a).

To identify patterns in national reimbursement criteria for bDMARD treatment, countries were assigned a composite eligibility score (see Table S2 in the supplementary material) and grouped into low-access (composite eligibility score 0–1), moderate-access (composite eligibility score 2–3), or high-access (composite eligibility score 4–5) clusters [11]. Most countries grouped into the same clusters (Figure 2(b,c)) as previously reported by Putrik and colleagues [11].

### Predicted bDMARD-eligible patient numbers defined by national reimbursement criteria

Using the criteria for disease severity and minimum csDMARD treatment failures, the nationally eligible proportion of the EULAR-defined population was calculated and the theoretical size of these patient populations was estimated (Figure 2(d)). It was found that the size of populations defined by national criteria were, for the most part, smaller than the populations defined by EULAR criteria.

**Figure 3.**
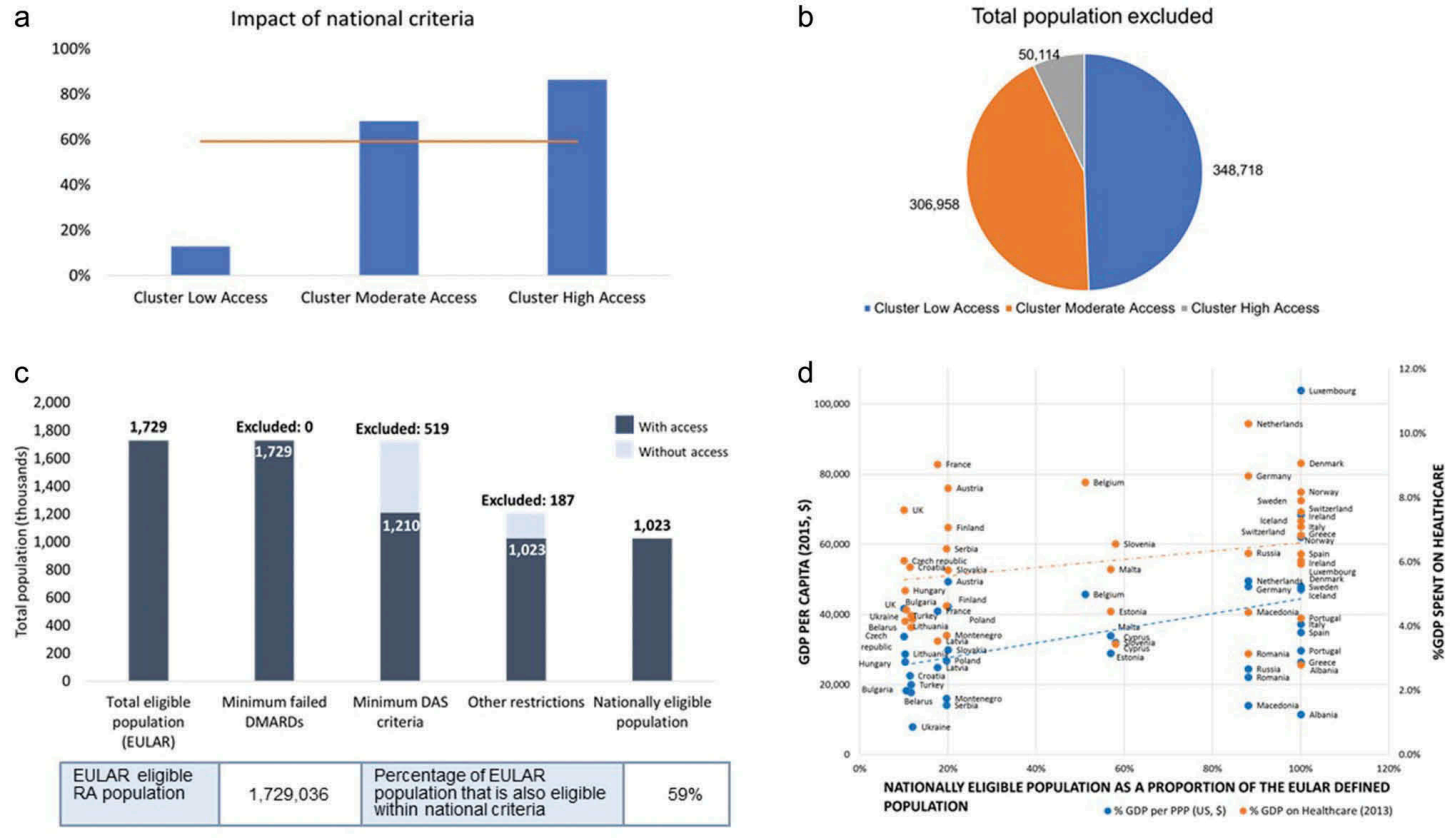
(A) Percentage of European League Against Rheumatism (EULAR)-eligible rheumatoid arthritis (RA) patients in each access group with access to biological disease-modifying antirheumatic drug (bDMARD) treatment on the basis of national criteria. The blue bars denote the eligible proportion of the population in each cluster. The horizontal line indicates the average eligible proportion of the total EULAR-eligible population. (B) Number of EULAR-eligible RA patients in each access group excluded from bDMARD treatment on the basis of national criteria for disease severity and minimum conventional synthetic DMARD treatment failures. (C) Numbers of EULAR-eligible RA patients who are excluded based on national criteria in the European region. In the graph, the dark blue bar denotes the RA population that is eligible according to the given criteria. DAS, Disease Activity Score. (D) The nationally defined RA population as a proportion of the EULAR-defined population plotted against gross domestic product (GDP) per capita in 2015 and % GDP spent on healthcare in 2013. PPP, purchasing power parity. The dotted lines indicate the positive linear correlation.

To summarize the differences across countries, the numeric impact of exclusion of EULAR-eligible RA patients on the basis of national reimbursement criteria was calculated for the low-, moderate-, and high-access clusters described above. In the high-access cluster 86% (318,085 nationally eligible patients out of the 368,199 total RA population of high-access countries), in the moderate-access cluster 68% (653,527 nationally eligible patients out of the 960,485 total RA population of moderate-access countries), and in the low-access cluster 13% (51,634 nationally eligible patients out of the 400,353 total RA population of low-access countries) of the EULAR-defined patient population are bDMARD eligible according to national reimbursement criteria (Figure 3(a,b)). On average, 59% of the EULAR-defined population is eligible for bDMARD treatment according to national criteria (Figure 3(c)).

### Relationship between access to bDMARD reimbursement and GDP or percentage GDP allocated to healthcare

The proportion of each country’s nationally eligible population, according to disease severity and minimum csDMARD treatment failures, as a percentage of the EULAR-defined population was compared with the country’s percentage GDP spent on healthcare in 2013 [18] and GDP per capita in 2015 [19] in a linear regression analysis, both of which revealed a weak positive correlation (*R*^2^ = 0.0684 and 0.1968, respectively), although slight improvement with the latter can be observed (Figure 3(d)).

## Discussion

This study sought to quantify the national reimbursement criteria for bDMARDs in terms of patient numbers, and found that across the European region, around 700,000 RA patients are excluded from reimbursed bDMARD treatment owing to national criteria that are more stringent than the recommendations made by EULAR. Exclusion from treatment is not uniform across Europe, as our survey of national reimbursement criteria for bDMARDs in the European region confirmed. Translating the reimbursement criteria into numbers, access to reimbursed bDMARDs in a given European country can range between 13% and 86% of a EULAR-defined RA population according to the model presented here. In contrast with Putrik and colleagues [11], who found an association between the number of restriction criteria applied to bDMARDs and GDP, a comparison of the actual patient numbers affected and percentage GDP per capita or health-care expenditure per GDP revealed only a slightly positive association.

Our approach has several limitations, notably that the severity of the patient population and point of RA diagnosis are assumed to be similar across all countries surveyed. Furthermore, mathematical estimates may exclude patients treated under research. An additional limitation in this study is that the accuracy of patient estimates relies on the quality of epidemiological data. Studies of RA prevalence are difficult to compare directly because of differences in methodology such as the age group included, and inclusion and diagnostic criteria. Nevertheless, genuine variations between different populations, such as the high prevalence of RA reported in the Netherlands and Belgium, have been suggested, and attributed to regional variation in behavioural factors, climate, environmental exposures, RA diagnosis, and genetic factors [20].

A comparison of the population model’s prediction of patient numbers with data from real-world settings was attempted (data not shown). However, the only published real-world studies were conducted at a time when bDMARDs were relatively new to the market and before publication of the 2013 EULAR guidelines [9,21,22]. In any case, comparisons with published real-world data have a number of limitations, including, most importantly, the diversity of data capture and extrapolation methodologies used within different sources of data. Many bDMARD treatments are used in more than one indication, making comparisons complex because estimates in the literature may make differential assumptions about bDMARD use within RA depending on the methodology chosen.

The patient numbers predicted by the population model provide a broad estimate of access to bDMARDs. However, it is to be expected that additional system characteristics, that is, unpublished factors that are intrinsic to individual healthcare systems, would define access to treatments such as bDMARDs even further. According to the results from the questionnaire, many countries in the European region have additional restrictions beyond the national reimbursement criteria for bDMARD eligibility. Local restrictions potentially influencing prescribers, rheumatology centres, patients, or pharmaceutical companies include limits on the number or quantity of bDMARDs that patients can be treated with (e.g. the maximum prescribed dose being 1 month of therapy and volume limits for individual prescribers or centres) and additional implementation burdens (e.g. complicated protocols, permits, and budget allocations) and process restrictions (e.g. strict prescriptions and required medical statements potentially necessitating long-distance travel to eligible rheumatology centres). Further research is needed to explore how these additional limitations influence patient access to reimbursed medicines.

The expected launch of biosimilars, alternatives to biological originators [23], is likely to generate significant savings for healthcare providers and may provide opportunities to improve access to current and future medicines for RA patients [24]. This study provides a snapshot of the RA patient populations that have access to reimbursed bDMARDs and provides a numeric platform for estimates of potential cost savings that could be generated upon the introduction of biosimilars, as well as estimates of patient populations that may be eligible in the future for bDMARD treatment pending revisions of national criteria.

Further detailed research into barriers to patient access beyond national reimbursement criteria and to the potential impact of biosimilar launches is warranted.

## Supplementary Material

Suppl_Tables.docx

## References

[1] Cross M, Smith E, Hoy D, et al The global burden of rheumatoid arthritis: estimates from the global burden of disease 2010 study. Ann Rheum Dis. 2014;73:1316–8. 24550173 10.1136/annrheumdis-2013-204627

[2] Furneri G, Mantovani LG, Belisari A, et al Systematic literature review on economic implications and pharmacoeconomic issues of rheumatoid arthritis. Clin Exp Rheumatol. 2012;30:S72–84. 23072761

[3] Brooks PM. The burden of musculoskeletal disease – a global perspective. Clin Rheumatol. 2006;25:778–781. 16609823 10.1007/s10067-006-0240-3

[4] Smolen JS, Landewé R, Breedveld FC, et al EULAR recommendations for the management of rheumatoid arthritis with synthetic and biological disease-modifying antirheumatic drugs: 2013 update. Ann Rheum Dis. 2014;73:492–509. 24161836 10.1136/annrheumdis-2013-204573 PMC3933074

[5] Singh JA, Saag KG, Bridges SL, et al American College of Rheumatology guideline for the treatment of rheumatoid arthritis. Arthritis Rheumatol (Hoboken, N.J.). 2015;68:1–26. 10.1002/art.39480 26545940

[6] Smolen JS, Landewe R, Bijlsma J, Burmester G, Chatzidionysiou K, Dougados M et al. Eular recommendations for the management of rheumatoid arthritis with synthetic and biological disease-modifying antirheumatic drugs: 2016 update. Ann Rheum Dis 2017; 76: 960–977. 28264816 10.1136/annrheumdis-2016-210715

[7] Aletaha D, Landewe R, Karonitsch T, et al Reporting disease activity in clinical trials of patients with rheumatoid arthritis: EULAR/ACR collaborative recommendations. Arthritis Rheum. 2008;59:1371–1377. 18821648 10.1002/art.24123

[8] Nam JL, Ramiro S, Gaujoux-Viala C, et al Efficacy of biological disease-modifying antirheumatic drugs: a systematic literature review informing the 2013 update of the EULAR recommendations for the management of rheumatoid arthritis. Ann Rheum Dis. 2014;73:516–528. 24399231 10.1136/annrheumdis-2013-204577

[9] Orlewska E, Ancuta I, Anic B, et al Access to biologic treatment for rheumatoid arthritis in central and Eastern European (CEE) countries. Med Sci Monit. 2011;17:SR1–13. 21455121 10.12659/MSM.881697 PMC3539513

[10] Putrik P, Ramiro S, Kvien TK, et al Inequities in access to biologic and synthetic DMARDs across 46 European countries. Ann Rheum Dis. 2014;73:198–206. 23467636 10.1136/annrheumdis-2012-202603

[11] Putrik P, Ramiro S, Kvien TK, et al Variations in criteria regulating treatment with reimbursed biologic DMARDs across European countries. Are differences related to country’s wealth? Ann Rheum Dis. 2014;73:2010–2021. 23940213 10.1136/annrheumdis-2013-203819

[12] Aletaha D, Smolen JS The rheumatoid arthritis patient in the clinic: comparing more than 1,300 consecutive DMARD courses. Rheumatology (Oxford). 2002;41:1367–1374. 12468815 10.1093/rheumatology/41.12.1367

[13] Humphreys JH, Verstappen SMM, Hyrich KL, et al The incidence of rheumatoid arthritis in the UK: comparisons using the 2010 ACR/EULAR classification criteria and the 1987 ACR classification criteria. Results from the Norfolk Arthritis Register. Ann Rheum Dis. 2013;72:1315–1320. 22945499 10.1136/annrheumdis-2012-201960 PMC3711368

[14] Sokka T, Kautiainen H, Toloza S, et al QUEST-RA: quantitative clinical assessment of patients with rheumatoid arthritis seen in standard rheumatology care in 15 countries. Ann Rheum Dis. 2007;66:1491–1496. 17412740 10.1136/ard.2006.069252 PMC2111618

[15] Kavanaugh A, Klareskog L, van der Heijde D, et al Improvements in clinical response between 12 and 24 weeks in patients with rheumatoid arthritis on etanercept therapy with or without methotrexate. Ann Rheum Dis. 2008;67:1444–1447. 18535115 10.1136/ard.2008.094524 PMC2566536

[16] Hetland ML, Christensen IJ, Tarp U, et al Direct comparison of treatment responses, remission rates, and drug adherence in patients with rheumatoid arthritis treated with adalimumab, etanercept, or infliximab: results from eight years of surveillance of clinical practice in the nationwide Danish DANIBO registry. Arthritis Rheum. 2010;62:22–32. 20039405 10.1002/art.27227

[17] World Bank World development indicators. Population. [Internet]. 2016. Available from: http://data.worldbank.org/indicator/SP.POP.TOTL

[18] World Bank Health expenditure, public (% of GDP). [Internet] 2013 Available from:http://data.worldbank.org/indicator/SH.XPD.PUBL.ZS

[19] World Bank GDP per capita, PPP (current international $). [Internet] 2015. Available from: ttp://data.worldbank.org/indicator/NY.GDP.PCAP.PP.CD?order

[20] Carmona L, Cross M, Williams B, et al Rheumatoid arthritis. Best Pract Res Clin Rheumatol. 2010;24:733–745. 21665122 10.1016/j.berh.2010.10.001

[21] European Medicines Agency (EMA) European public assessment reports [Internet]. [cited 2017 6 6]. Available from: http://www.ema.europa.eu/ema/index.jsp?curl=pages%2Fmedicines%2Flanding%2Fepar_search.jsp&mid=WC0b01ac058001d124&searchTab=&alreadyLoaded=true&isNewQuery=true&status=Authorised&keyword=Enter+keywords&searchType=name&taxonomyPath=Diseases.Musculoskeletal+D

[22] Kobelt G, Lekander I, Santesson Nicolae Y Access to innovative treatments for rheumatoid arthritis in New Zealand. A comparison with Australia and the UK. [Internet] 2010 Available from : https://www.arthritis.org.nz/wp-content/uploads/2014/05/ACCESS-TO-INNOVATIVE-TREATMENTS-RA-FINAL.pdf

[23] World Health Organization Guidelines on evaluation of similar biotherapeutic products (SBPs) [Internet]. 2009 Available from: http://www.who.int/biologicals/areas/biological_therapeutics/BIOTHERAPEUTICS_FOR_WEB_22APRIL2010.pdf

[24] Gulácsi L, Brodszky V, Baji P, et al Biosimilars for the management of rheumatoid arthritis: economic considerations. Expert Rev Clin Immunol. 2015;11(Suppl 1):S43–52. 26395836 10.1586/1744666X.2015.1090313

