# Supplementary material for: Patient access to reimbursed biological disease-modifying antirheumatic drugs in the European region
Source: J Mark Access Health Policy. 2017 Jul 5;5(1):1345580. doi: 10.1080/20016689.2017.1345580 (PMC5508389; doi:10.1080/20016689.2017.1345580)
Supplement: Suppl_Tables.docx [file zjma_a_1345580_sm1480.docx]

# Supplementary Tables

## Accompanying manuscript

“Patient access to reimbursed biologic disease modifying antirheumatic drugs in the European region”

Table S1 Population sizes (from World Bank Group. [2014])[1] and prevalence of rheumatoid arthritis (RA) and size of RA patient population

| Country | Population | RA Prevalence | Prevalent RA population | Reference |
| --- | --- | --- | --- | --- |
| Albania | 2894475 | 0.34% | 9841.215 | Koko V et al. 2015[2] |
| Austria | 8534492 | 0.83% | 70836.2836 | Taken from Germany |
| Belarus | 9480900 | 0.55% | 52144.95 | Taken from Lithuania |
| Belgium | 11225207 | 3.14% | 352471.4998 | Taken from the Netherlands |
| Bulgaria | 7226291 | 0.62% | 44803.0042 | Taken from Czech |
| Croatia | 4236400 | 0.35% | 14827.4 | Taken from Serbia |
| Cyprus | 1153658 | 0.68% | 7844.8744 | Taken from Greece |
| Czech Republic | 10510566 | 0.62% | 65165.5092 | Hanova P et al. 2006[3] |
| Denmark | 5639565 | 0.75% | 42296.7375 | Pedersen J et al. 2011[4] |
| Estonia | 1313645 | 0.44% | 5780.038 | Otsa K et. al. 2013[5] |
| Finland | 5463596 | 0.70% | 38245.172 | Aho K et al. 1989[6] |
| France | 66206930 | 0.32% | 211862.176 | Guillemin F et al 1994 [7] |
| Germany | 80889505 | 0.83% | 671382.8915 | Taken from UK |
| Greece | 10957740 | 0.68% | 74512.632 | Andrianakos A et al. 2006[8] |
| Hungary | 9861673 | 0.37% | 36488.1901 | Kiss C et al. 2005[9] |
| Iceland | 327589 | 0.47% | 1539.6683 | Taken from Norway |
| Ireland | 4612719 | 0.50% | 23063.595 | Power Det al. 1999 [10] |
| Italy | 61336387 | 0.33% | 202410.0771 | Cimmino M et al. 1998 [11] |
| Latvia | 1990351 | 0.44% | 8757.5444 | Taken from Estonia |
| Lithuania | 2929323 | 0.55% | 16111.2765 | Adomaviciute D et al. 2008 [12] |
| Luxembourg | 556074 | 0.32% | 1779.4368 | Taken from France |
| Macedonia | 2075625 | 0.35% | 7264.6875 | Taken from Serbia |
| Malta | 427404 | 0.68% | 2906.3472 | Taken from Greece |
| Montenegro | 621800 | 0.35% | 2176.3 | Taken from Serbia |
| Netherlands | 16854183 | 3.14% | 529221.3462 | Picavet H et al. 2003[13] |
| Norway | 5136475 | 0.47% | 24141.4325 | Riise T et al. 2000[14] |
| Poland | 37995529 | 0.62% | 235572.2798 | Taken from Czech Republic |
| Portugal | 10397393 | 0.50% | 51986.965 | Monjardino T et al. 2011[15] |
| Romania | 19910995 | 0.62% | 123448.169 | Taken from Czech Republic |
| Russia | 142098141 | 0.61% | 866798.6601 | Galushko et al 2010[16] |
| Serbia | 7129428 | 0.35% | 24952.998 | Zlatković-Švenda M et al. 2014[17] |
| Slovakia | 5418506 | 0.35% | 18964.771 | Taken from Serbia |
| Slovenia | 2062218 | 0.35% | 7217.763 | Taken from Serbia |
| Spain | 46404602 | 0.52% | 241303.9304 | Carmona L et al 2001[18]; Carmona L et al 2002[19] |
| Sweden | 9689555 | 0.70% | 67826.885 | Neovius M et al 2011[20] |
| Switzerland | 8190229 | 0.32% | 26208.7328 | Taken from France |
| Turkey | 75932348 | 0.49% | 372068.5052 | Akar S et al. 2004[21] |
| UK | 64510376 | 0.83% | 535436.1208 | Symmons D et al 2002 [22] |
| Ukraine | 42500501 | 0.61% | 259253.0561 | Taken from Russia |

Table S2 National reimbursement criteria for biologic DMARDs in countries of the European region according to expert responses. Composite scores according to Putrik and colleagues (2014)[23].

| Country | Minimum  disease  duration | Disease activity | Number of csDMARDs failed | Type of  previous  treatment | Time to evaluate biologic DMARD response | Stop Criteria | Switch Criteria | Compo-  site score |
| --- | --- | --- | --- | --- | --- | --- | --- | --- |
| Albania | None | DAS28 > 4.5 | 2 DMARDs | MTX | 12 to 24 weeks | None | None | 2 |
| Austria | None | Moderate to severe active RA | 1 DMARD | MTX | None | None | None | 4 |
| Belarus | None | DAS28 > 5.1 | 2 DMARDs | MTX and SSF or LEF | 12 weeks | Improvement DAS28 < 1.2 | Improvement DAS28 < 1.2 | 2 |
| Belgium | None | DAS28 > 3.7 | 2 DMARDs | MTX | 24 weeks | DAS28 > 5.1 and improvement < 1.2  or DAS28 < 5.1 and improvement < 0.6 | None | 2 |
| Bulgaria | 6 months | DAS28 > 5.1 | 2 DMARDs | MTX and SSF or LEF | 12 weeks | None | Improvement DAS28 ≤ 0.6 | 1 |
| Croatia | 6 months | DAS28≥5.1 or DAS28 ≥ 3.2 + 6 swollen joints (of 44), HAQ 0.5-2.5 | 2 DMARDs | MTX | 12 weeks | CRP ≤1 mg/dL; Number of painful joints /28≤1; Number of swollen joints /28≤1; HAQ (0-10) ≤1) | None | 1 |
| Cyprus | None | DAS28 > 4 | 2 DMARDs | MTX | 12 weeks | DAS28 > 3.2 and improvement <1.2 | DAS28 ˃ 3.2, improvement DAS28 < 1.2 | 5 |
| Czech Republic | 6 months | DAS28 > 5.1 | 1 DMARD | Any sDMARD | 12 weeks | Improvement DAS28 < 1.2 | None | 2 |
| Denmark | 3 months | DAS28 > 3.2 or Radiographic progression | 3 DMARDs | MTX & Prednisone | 12 to 16 weeks | None | None | 3 |
| Estonia | 6 months | DAS28 > 4.6 and TJO > 8 and SJC > 6 + Other factors | 4 DMARDs | MTX | 12 to 24 weeks | Improvement DAS28 < 1.2 | DAS28 >4.6 and TJO>8 and SJC>6 + other factors | 0 |
| Finland | None | DAS28 > 5.1 | 3 DMARDs | None | None | None | None | 2 |
| France | None | DAS28 > 5.1 | No DMARD restriction | None | 12 weeks | None | None | 3 |
| Germany | None | None | 2 DMARDs | MTX and SSF or LEF | 12 weeks | None | None | 5 |
| Greece | None | DAS28 > 3.2 + prognostic factors  or DAS28 >5.1 | 1 to 2 DMARDs | MTX and LEF or SSF, HCQ, AZT, Cyclos-A | 16 weeks | DAS28 >3.2 or failure to achieve remission (DAS28 <2.6) | DAS28 >3.2 or failure to achieve remission (DAS28 <2.6) | 3 |
| Hungary | None | DAS28 > 5.1 | No DMARD restriction | MTX | 12 weeks | Improvement DAS28 < 1.2 | Improvement DAS28 < 1.2 | 3 |
| Iceland | None | DAS28 > 3.2 | 2 DMARDs | MTX and SSF or LEF | 12 to 24 weeks | None | DAS28 > 3.2 | 4 |
| Ireland | None | None | 1 DMARD | MTX | 12 weeks | None | None | 5 |
| Italy | 3 months | DAS28 >3.2 | 1 DMARD | MTX | None | None | None | 3 |
| Latvia | None | DAS28 > 5.1 | 2 DMARDs | MTX | 12 weeks | None | DAS28 > 3.2 | 2 |
| Lithuania | None | DAS28 > 5.1 | 2 DMARDs | MTX and SSF or LEF | 12 weeks | Improvement DAS28 < 1.2 | Improvement DAS28 < 1.2 | 2 |
| Luxembourg | None | None | No DMARD restriction | None | None | None | None | 5 |
| Macedonia | None | DAS28 >4.2 and SJC≥6 | 2 DMARDs | MTX | 6 to 12 weeks | None | None | 2 |
| Malta | 6 months | None | 2 DMARDs | MTX and SSF or LEF | 12 weeks | DAS28 > 3.2 and improvement <1.2 | DAS28 ˃ 3.2, improvement DAS28 < 1.2 | 1 |
| Montenegro | 6 months | DAS28 > 5.1 and HAQ 1-2.5 + other factors | 2 DMARDs | MTX and SSF or LEF | 12 weeks | None | DAS28 ˃ 3.2, improvement DAS28 < 1.2 | 1 |
| Netherlands | None | DAS28 > 3.2 | 2 DMARDs | MTX and SSF or LEF | 24 weeks | None | Improvement DAS28 < 1.2 | 3 |
| Norway | None | DAS28 > 3.2 | 1 DMARD | None | 12 to 24 weeks | None | None | 4 |
| Poland | 12 months | DAS28 > 5.1 or >3.7 or SDAI >26 | 2 DMARDs | MTX | 13 weeks | DAS28 < 3,2; DAS < 2,4; SDAI < 11 at 6 months | None | 1 |
| Portugal | None | DAS28 > 3.2 or DAS28 2.6-3.2 and worsening of HAQ > 0.22 or X-ray scores | 1 DMARD | MTX | 12 weeks | Improvement DAS28 < 2.6 | Improvement DAS28 < 1.2 | 4 |
| Romania | None | DAS28 >3.2; DAS28 > 5.1 including 5 joints with active synovitis and other factors | 2 DMARDs | MTX and LEF or SSF, HCQ, AZT, Cyclos-A | 24 weeks | According to EULAR | According to EULAR | 2 |
| Russia | None | None | 3 DMARDs | MTX and LEF or SSF, HCQ, AZT, Cyclos-A | 24 weeks | None | None | 3 |
| Serbia | 6 months | DAS28 > 5.1 and HAQ 1-2.5 | No DMARD restriction | MTX and SSF or LEF | 12 weeks | None | DAS28 ˃ 3.2, improvement DAS28 < 1.2 | 1 |
| Slovakia | None | DAS28 > 5.1 or >3.7 | 1 DMARD | MTX | 12 weeks | None | DAS28 > 0.6 and ≤ 1.2 | 4 |
| Slovenia | None | DAS28≥ 3,2 and SJC>8 swollen and painful joints (of 44), Sedimentation and VAS | 2 DMARDs | MTX | 24 weeks | DAS28 > 3.2 and improvement <1.2 | None | 3 |
| Spain | None | DAS28 ≥3.2 or SDA ≥11 or  DAS28 2.6-3.2 or SDA 5-11 and prognostic indicators | No DMARD restriction | None | 12 weeks | None | DAS28 ≥3.2 or SDA ≥11 or  DAS28 2.6-3.2 or SDA 5-11 and prognostic indicators | 4 |
| Sweden | None | DAS28 > 3.2 + prognostic factors or DAS28 >5.1 | No DMARD restriction | None | 12 weeks | None | DAS28 > 3.2 | 3 |
| Switzerland | None | None | 1 DMARD | None | None | None | None | 5 |
| Turkey | None | DAS28 > 5.1 | 3 DMARDs | MTX | 12 weeks | Improvement DAS28 < 1.2 | None | 1 |
| UK | 6 months | DAS28 > 5.1 | 2 DMARDs | MTX | 26 weeks | Improvement DAS28 < 1.2 | Improvement DAS28 < 1.2 | 1 |
| Ukraine | 6 months | DAS28 > 5.3 | 1 DMARD | Any csDMARD | 12 weeks | Improvement DAS28 ≤ 0.6 | Improvement DAS28 ≤ 0.6 | 2 |

**Abbreviations:** AZT, Zidovudine; Cyclos-A, Cyclosporine-A; DAS, Disease Activity Score; DMARD, disease-modifying antirheumatic drug; EULAR, European League Against Rheumatism; HCQ, Hydroxychloroquine; LEF, Leflunomide; MTX, Methotrexate; SSF, Sulfasalazine;

References

[1] World Bank. World Development Indicators n.d.

[2] Koko V, Ndrepepa A, Skenderaj S. Epidemiology of Rheumatoid Arthritis in Southern Albania. Mater Sociomed 2015;27:172–5. doi:10.5455/msm.2015.27.172-175.

[3] Hanova P, Pavelka K, Dostal C, Holcatova I, Pikhart H. Epidemiology of rheumatoid arthritis, juvenile idiopathic arthritis and gout in two regions of the Czech Republic in a descriptive population-based survey in 2002-2003. Clin Exp Rheumatol 2006;24:499–507.

[4] Pedersen JK, Svendsen AJ, Hørslev-Petersen K. Prevalence of rheumatoid arthritis in the southern part of denmark. Open Rheumatol J 2011;5:91–7. doi:10.2174/1874312901105010091.

[5] Otsa K, Tammaru M, Vorobjov S, Esko M, Pärsik E, Lang K. The prevalence of rheumatoid arthritis in Estonia: an estimate based on rheumatology patients’ database. Rheumatol Int 2013;33:955–8. doi:10.1007/s00296-012-2473-6.

[6] Aho K, Heliövaara M, Sievers K, Maatela J, Isomäki H. Clinical arthritis associated with positive radiological and serological findings in Finnish adults. Rheumatol Int 1989;9:7–11.

[7] Guillemin F, Briançon S, Klein JM, Sauleau E, Pourel J. Low incidence of rheumatoid arthritis in France. Scand J Rheumatol 1994;23:264–8.

[8] Andrianakos A, Trontzas P, Christoyannis F, Kaskani E, Nikolia Z, Tavaniotou E, et al. Prevalence and management of rheumatoid arthritis in the general population of Greece--the ESORDIG study. Rheumatology (Oxford) 2006;45:1549–54. doi:10.1093/rheumatology/kel140.

[9] Kiss CG, Lövei C, Sütö G, Varjú C, Nagy Z, Füzesi Z, et al. Prevalence of rheumatoid arthritis in the South-Transdanubian region of Hungary based on a representative survey of 10,000 inhabitants. J Rheumatol 2005;32:1688–90.

[10] Power D, Codd M, Ivers L, Sant S, Barry M. Prevalence of rheumatoid arthritis in Dublin, Ireland: a population based survey. Ir J Med Sci 1999;168:197–200.

[11] Cimmino MA, Parisi M, Moggiana G, Mela GS, Accardo S. Prevalence of rheumatoid arthritis in Italy: the Chiavari Study. Ann Rheum Dis 1998;57:315–8.

[12] Adomaviciute D, Pileckyte M, Baranauskaite A, Morvan J, Dadoniene J, Guillemin F. Prevalence survey of rheumatoid arthritis and spondyloarthropathy in Lithuania. Scand J Rheumatol 2008;37:113–9. doi:10.1080/03009740701774966.

[13] Picavet HSJ, Hazes JMW. Prevalence of self reported musculoskeletal diseases is high. Ann Rheum Dis 2003;62:644–50.

[14] Riise T, Jacobsen BK, Gran JT. Incidence and prevalence of rheumatoid arthritis in the county of Troms, northern Norway. J Rheumatol 2000;27:1386–9.

[15] Monjardino T, Lucas R, Barros H. Frequency of rheumatic diseases in Portugal: a systematic review. Acta Reum Port 2011;36:336–63.

[16] Galushko EA, Erdes SF, Bazorkina DI, Bol’shakova TI, Vinogradova IB, Lesniak OM, et al. [Prevalence of rheumatoid arthritis in Russia (according to epidemiological findings)]. Ter Arkhiv 2010;82:9–14.

[17] Zlatković-Švenda MI, Stojanović RM, B Šipetić-Grujičić S, Guillemin F. Prevalence of rheumatoid arthritis in Serbia. Rheumatol Int 2014;34:649–58. doi:10.1007/s00296-013-2897-7.

[18] Carmona L, Ballina J, Gabriel R, Laffon A. The burden of musculoskeletal diseases in the general population of Spain: results from a national survey. Ann Rheum Dis 2001;60:1040–5.

[19] Carmona L, Villaverde V, Hernández-García C, Ballina J, Gabriel R, Laffon A. The prevalence of rheumatoid arthritis in the general population of Spain. Rheumatology (Oxford) 2002;41:88–95.

[20] Neovius M, Simard JF, Askling J. Nationwide prevalence of rheumatoid arthritis and penetration of disease-modifying drugs in Sweden. Ann Rheum Dis 2011;70:624–9. doi:10.1136/ard.2010.133371.

[21] Akar S, Birlik M, Gurler O, Sari I, Onen F, Manisali M, et al. The prevalence of rheumatoid arthritis in an urban population of Izmir-Turkey. Clin Exp Rheumatol 2004;22:416–20.

[22] Symmons D, Turner G, Webb R, Asten P, Barrett E, Lunt M, et al. The prevalence of rheumatoid arthritis in the United Kingdom: new estimates for a new century. Rheumatology (Oxford) 2002;41:793–800.

[23] Putrik P, Ramiro S, Kvien TK, Sokka T, Uhlig T, Boonen A. Variations in criteria regulating treatment with reimbursed biologic DMARDs across European countries. Are differences related to country’s wealth? Ann Rheum Dis 2014;73:2010–21. doi:10.1136/annrheumdis-2013-203819.
